# An Overview on the Physiopathology of the Blood–Brain Barrier and the Lipid-Based Nanocarriers for Central Nervous System Delivery

**DOI:** 10.3390/pharmaceutics16070849

**Published:** 2024-06-22

**Authors:** Francesca Susa, Silvia Arpicco, Candido Fabrizio Pirri, Tania Limongi

**Affiliations:** 1 Department of Applied Science and Technology, Politecnico di Torino, Corso Duca degli Abruzzi 24, 10129 Turin, Italy; francesca.susa@polito.it (F.S.); fabrizio.pirri@polito.it (C.F.P.); 2 Department of Drug Science and Technology, University of Turin, Via Pietro Giuria 9, 10125 Turin, Italy; silvia.arpicco@unito.it

**Keywords:** blood–brain barrier, brain diseases, nanotechnologies, liposomes, extracellular vesicles, lipid-based nanocarriers, drug delivery

## Abstract

The state of well-being and health of our body is regulated by the fine osmotic and biochemical balance established between the cells of the different tissues, organs, and systems. Specific districts of the human body are defined, kept in the correct state of functioning, and, therefore, protected from exogenous or endogenous insults of both mechanical, physical, and biological nature by the presence of different barrier systems. In addition to the placental barrier, which even acts as a linker between two different organisms, the mother and the fetus, all human body barriers, including the blood–brain barrier (BBB), blood–retinal barrier, blood–nerve barrier, blood–lymph barrier, and blood–cerebrospinal fluid barrier, operate to maintain the physiological homeostasis within tissues and organs. From a pharmaceutical point of view, the most challenging is undoubtedly the BBB, since its presence notably complicates the treatment of brain disorders. BBB action can impair the delivery of chemical drugs and biopharmaceuticals into the brain, reducing their therapeutic efficacy and/or increasing their unwanted bioaccumulation in the surrounding healthy tissues. Recent nanotechnological innovation provides advanced biomaterials and ad hoc customized engineering and functionalization methods able to assist in brain-targeted drug delivery. In this context, lipid nanocarriers, including both synthetic (liposomes, solid lipid nanoparticles, nanoemulsions, nanostructured lipid carriers, niosomes, proniosomes, and cubosomes) and cell-derived ones (extracellular vesicles and cell membrane-derived nanocarriers), are considered one of the most successful brain delivery systems due to their reasonable biocompatibility and ability to cross the BBB. This review aims to provide a complete and up-to-date point of view on the efficacy of the most varied lipid carriers, whether FDA-approved, involved in clinical trials, or used in in vitro or in vivo studies, for the treatment of inflammatory, cancerous, or infectious brain diseases.

## 1. Introduction

One of the main functions of the vascular system is to deliver oxygen and nutrients from the heart to all functional districts and, at the same time, remove carbon dioxide and metabolic waste from tissues. This system is composed of arteries and arterioles, which deliver blood to the tissues of the capillary bed and assist gas and nutrient exchange within tissues, and venules and veins, which drain blood from tissues. The microvasculature, comprising capillaries and postcapillary venules, is the constitutive component for the establishment and maintenance of tissue health via blood perfusion and a dynamic interaction between tissues and the extracellular environment [1]. 

The unique microvasculature system present in the brain is the blood–brain barrier (BBB). Its peculiarity relies on the presence of continuous non-fenestrated capillaries, allowing at the same time precise regulation of brain homeostasis and protection from physical, chemical, and biological external agents. 

However, although the strict selectivity of BBB is necessary for proper brain functioning, it undoubtedly represents an obstacle to the effective delivery of drugs in the case of neurodegenerative diseases or cancer, excluding more than 98% of therapeutic molecules from entering the brain [2]. This BBB feature makes the treatments of many brain diseases, such as brain tumors, Alzheimer’s disease (AD), Parkinson’s disease (PD), and Huntington’s disease (HD), difficult and sometimes ineffective.

There are different methods to increase the efficacy of drug delivery across the BBB, but each of them has some disadvantages. The lipid solubility of the drug can be improved, but it can also affect its pharmacological activity. The BBB could be temporarily and reversibly disrupted in a non-specific manner and could either damage endothelial cells or brain tissues, allowing at the same time the access of harmful or toxic compounds. The BBB could also be bypassed through the intranasal route, limiting drug administration, and by means of invasive approaches, causing patient discomfort and possible sites of pathogen entry [3]. 

However, new targeted drug delivery approaches based on inorganic and organic nanoparticles (NPs) use provide a systemic brain-targeted administration with limited off-target effects and damages to the BBB. Inorganic NPs are non-degradable, have intrinsic toxicity, and are frequently used as contrast agents in imaging. Organic NPs characterized by higher biocompatibility, lower toxicity, and extensive loading and functionalization possibilities are often employed as nanocarriers for BBB crossing.

This field develops very rapidly, and in this updated review, we give an overview of the BBB structure and physiopathology, shedding light on the universally recognized mechanisms regarding the different ways to approach this barrier. Among the various strategies developed to deliver drugs to the brain, besides methods that use ligand conjugation for active targeting and techniques allowing temporary BBB disruption, many recent nanotechnological solutions have been designed to enhance the efficacy of the available pharmacological treatments for brain diseases. Many nanocarriers have been designed and tested as central nervous system (CNS) delivery systems, both for diagnosis and/or therapy [4,5,6], and in this review, we focused our attention on the lipid-based ones. Nanocarriers such as liposomes, solid lipid nanoparticles (SLNs), nanoemulsions (NEs), nanostructured lipid carriers (NLCs), niosomes, proniosomes, cubosomes, extracellular vesicles (EVs), and cell membrane-derived nanocarriers were described. For each of these categories of lipid nanocarriers, a table containing the experimental studies published from 2016 to date referring to specific brain diseases for both in vitro and in vivo studies was reported. For each nanocarrier, we combined a detailed description not only regarding its therapeutic cargoes but also all the functionalization methods and/or solutions applied to target the brain, highlighting the nanocarriers that have already undergone clinical trials.

## 2. The Blood–Brain Barrier

Between the end of the 19th century and the beginning of the 20th century, microbiologists Paul Ehrlich and his student Edwin Goldmann observed during histological labeling experiments that when a dye is injected systemically, it does not reach the brain, while if injected in the cerebrospinal fluid (CSF), it does not spread to the other organs. However, in 1898, Max Lewandowsky was the first researcher to postulate the existence of a specialized barrier in the brain; thus, he coined the term BBB. Only in the late 1960s did Reese and Karnovsky visualize the BBB during electron microscopy experiments [7,8].

The BBB strictly controls the permeability of cerebral capillaries and, selectively filtering what should enter the brain and what should not, ensures that the right concentrations of ions, amino acids, and peptides are maintained, preserving the homeostasis of the brain microenvironment [9]. 

### 2.1. BBB Structure

**Figure 1:**
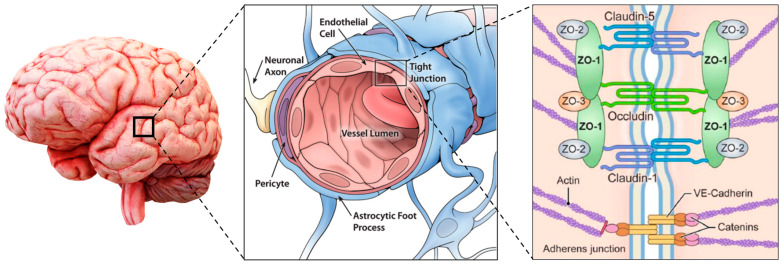
Scheme of the BBB neurovascular unit, modified from [10,11].

The physiological filtering properties of the BBB are conferred by the interactions between different cell lines: those constituting the blood vessels, endothelial (ECs) and mural (MCs) cells, glial and neural cells, and those of the immune system. The structure of the BBB is represented in Figure 1.

#### 2.1.1. Endothelial Cells and Junctions

The endothelial cells are squamous epithelial cells forming the walls of the vessels, and, in the central nervous system, they are phenotypically different from the ones located in other parts of the body. They have a luminal/abluminal polarization and tightly regulate the ions, molecules, and cell exchange through the tight junctions (TJs), which strictly limit the paracellular flux of solutes. In addition, CNS ECs have extremely low rates of transcytosis if compared with other ECs, greatly restricting vesicle-mediated transcellular transport. CNS ECs have peculiar features that could be found only in BBB and allow them to tightly regulate CNS homeostasis. In detail, they express efflux transporters for lipophilic molecules and highly specific transporters for the conveyance of nutrients and the removal of waste products. 

If compared to ECs from other tissues, CNS ECs have a higher number of mitochondria to generate the adenosine triphosphate (ATP) needed for transport functions, low levels of leukocyte adhesion molecules to limit immune cell entry, and a different metabolism to alter the physical properties of molecules, changing their reactivity, solubility, and transport properties [12]. 

ECs are sealed in their conjunction sites by different types of junctions. Tight junctions, which include integral membrane proteins such as claudin, occludin, junction adhesion molecules, and various cytoplasmic accessory proteins, are close to the apical membrane and limit the paracellular diffusion of solutes across the BBB [13]. TJs proteins are connected to the actin and vinculin-based cytoskeletal filaments via scaffolding proteins of the membrane-associated guanylate kinase family zonula occludens (ZO)-1, -2, and -3 [14].

TJs are stabilized by adherens junctions (AJs), which are close to the basolateral membrane and comprise cadherin, catenin, alpha-actinin, and vinculin, forming homophilic endothelial-to-endothelial contacts roughly 20 nm wide and participating in the development and preservation of TJs [15]. AJs are connected to the EC cytoskeleton, modulate receptor signaling, and regulate the transendothelial migration of immune cells [14]. AJs are crucial for the integrity of TJs, and their damage leads to disruption of the BBB. The reduction in TJs increases the probability of tumor metastasis since they are on the frontlines as the structure that cancer cells must overcome to metastasize [16]. 

In addition to the previous ones, there are also the gap junctions, which include connexin-37, 40, and 43, and they establish hemichannels between ECs, allowing endothelial intercellular communications, but also maintaining the TJs integrity [14]. 

The restrictions on the paracellular movement of ions and charged molecules cause a high transendothelial electrical resistance measurable across the BBB [17].

#### 2.1.2. Basement Membrane

The basement membrane (BM) surrounds the blood vessels and can be divided into the inner vascular BM, secreted by ECs and pericytes (PCs), and the outer parenchymal BM, secreted by astrocytes. It is composed of different molecules, such as type IV collagens, laminin, nidogen, heparin sulfate proteoglycans, and other glycoproteins. Besides the support function, the BM also acts as an additional barrier. During different neurological diseases, BM is impaired by matrix metalloproteinases, leading to leakage in its barrier functions. 

#### 2.1.3. Pericytes

Pericytes are contractile MCs that partially wrap around the abluminal surface of ECs with their long cellular processes and are included in the BM. They can tune the diameter of the capillary and thus the blood flow in response to neural activity, thanks to contractile proteins. They also play a key role in angiogenesis, deposition of extracellular matrix, wound healing, and immune cell infiltration. They closely interact with ECs, and a disruption of these interactions may lead to BBB dysfunction and neuroinflammation. 

#### 2.1.4. Astrocytes

The major type of glial cells in the BBB are astrocytes (ACs), and their end-feet, surrounding ECs, BMs, and PCs, provide a link between the neuronal circuitry and the bloodstream. 

Moreover, ASs can also increase the level of TJ proteins and inhibit the differentiation of pericytes, essential functions to maintain BBB integrity and low permeability. 

In the BBB, there are different types of ACs, depending on their morphology, origin, density, and function, and eventually adapting to the needs of the microenvironment. The most abundant types are protoplasmic astrocytes in the gray matter, with many radially extending processes, and fibrous astrocytes in the white matter, with smoother and longer processes [18].

#### 2.1.5. Microglia

Microglia are monocyte-resident cells throughout the brain and spinal cord. Being the resident macrophage cells, their main functions are immune defense and CNS preservation, but they can also modulate the expression of TJs [19].

### 2.2. BBB Transport Mechanisms

**Figure 2:**
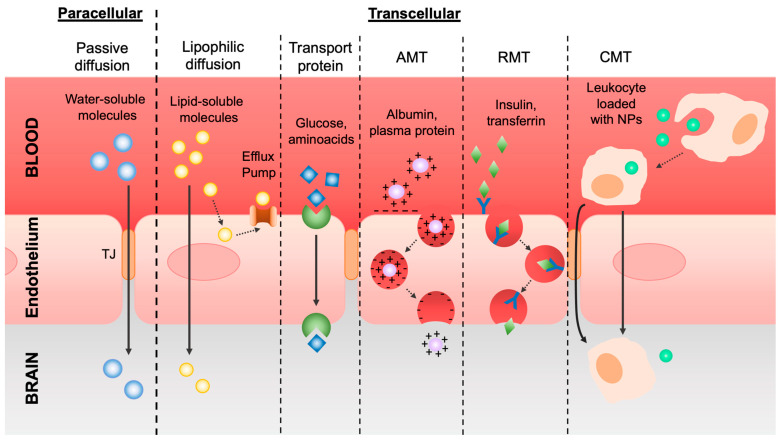
The different molecules’ transport mechanisms in the BBB are divided into paracellular and transcellular.

Flow across the BBB is regulated through different transport mechanisms (Figure 2). 

The first mechanism of transport is the one referred to as the diffusion of molecules via the paracellular or transcellular pathways. Small water-soluble molecules can cross the BBB through paracellular passive diffusion following the negative gradient of concentration across the TJs. In addition, the presence of particular enzymes in the abluminal part of the vessels causes the degradation of unwanted small molecules that eventually infiltrate by this mechanism. Lipophilic, non-polar, and low-molecular-weight molecules, such as oxygen and carbon dioxide, but also alcohol and anesthetics, can cross the membranes of the ECs, entering the BBB via a transcellular pathway. 

To eventually remedy harmful lipophilic molecules permeation, on the membranes of ECs there are efflux pumps able to drain these substances out of the cerebral tissue into the bloodstream. The most important one is the active drug efflux transporter of the ATP-binding cassette (ABC) gene family, which is notably responsible for drug distribution and elimination from the CNS and is understandably one of the major obstacles to effective drug delivery to the brain [20].

In contrast, polar and high-molecular-weight molecules, such as glucose and amino acids, which cannot easily cross the BBB through passive diffusion, exploit transport proteins or carriers. These types of solutes bind to a transporter on the luminal side of the EC membrane and, triggering a conformational change in the protein, are released by the abluminal side in the brain. This active transport depends on Na^+^ gradients such as sodium-dependent glucose transporters and amino acid transporters of glutamate and aspartate [21]. 

Regarding ions, their permeability is driven by an electrostatic interaction between the macromolecules’ positive charge and the negatively charged membranes, following a pathway called adsorptive-mediated transcytosis (AMT), or pinocytosis. Cationic molecules, such as cationized albumin and cell-penetrating peptides, bind to the luminal surface of ECs and are then exocytozed at the abluminal surface [22].

Another type of transcytosis is receptor-mediated transcytosis (RMT), for the selective delivery of macromolecules, including transferrin, melanotransferrin, insulin, leptin, TNF-alpha, and epidermal growth factor. In detail, the specific macromolecules (ligands) bind to the specific receptors in clathrin-coated pits, specialized areas of the plasma membrane. Then, these coated pits invaginate into the cytoplasm, forming coated vesicles. The ligand can dissociate from the receptor once the acidification of the endosome is complete and cross to the other side of the membrane [20,23]. 

In addition to these mechanisms, there is also cell-mediated transcytosis (CMT), which is usually exploited by pathogens to enter the CNS but can also be exploited for drug transport. In brief, pathogens or drugs can be easily phagocytized by leukocytes and then cross the BBB through diapedesis and chemotaxis. The infiltration of the immune cells is a dynamic and complex procedure that requires a series of stages such as tethering, rolling, crawling, arrest, and diapedesis across the ECs. However, in pathological conditions, the TJs among ECs may be disturbed by cytokines and other proinflammatory factors, letting macrophages and monocytes enter the brain by paracellular and transcellular pathways [12,24].

## 3. Brain Diseases

Besides the recent advancement in medicine, brain diseases remain one of the most important causes of death, health loss, and disability worldwide; according to the World Health Organization, 3.4 billion people are affected by neurological pathologies or disorders [25]. The difficulty of treatment of brain diseases mainly relies on their heterogeneity, the lack of proper preclinical models, and, overall, the presence of the BBB, which rejects more than 98% of the substances used for therapeutic treatment [26].

The role of the BBB is key for the diagnosis and especially for the treatment of brain diseases. Its integrity is physiologically related to the health status and the age of the patients, and it could be directly impaired by some brain diseases, such as stroke, tumors, or neurodegenerative diseases. An impaired BBB can cause an alteration to brain homeostasis, such as ion imbalance and the entry of immune cells and molecules, potentially leading to neuronal dysfunction and degeneration [1]. Many studies have shown that plasma proteins can be neurotoxic, suggesting that even if a compromised BBB does not cause these disorders, it can exacerbate them [13]. Furthermore, BBB, by using standard drug delivery administration methods, mechanically and biochemically prevents the efficient delivery of therapeutics in the brain to the injured sites, and, if locally delivered, its presence notably limits the diffusion of the active molecules. In the following paragraphs, we give an overview of the most diffused brain pathologies.

### 3.1. Stroke 

Stroke is one of the most common causes of adult disability and/or death. After an ischemic or hemorrhagic stroke, the intense neuroinflammation unleashes a cascade of events, such as acute BBB breakdown, cytotoxic and vasogenic edema, and hemorrhagic transformation, to remove the damaged tissue and prepare the brain for repair. It also contributes to neuronal injury and worsens neurological outcomes. Besides, in an early phase, neuroinflammation causes brain damage; it could later promote recovery by facilitating neurogenesis, angiogenesis, and neuronal plasticity [27]. 

A cerebral stroke leads to a hypoxic state, causing an increase in BBB permeability and TJ alterations. This increased permeability could be continuous, monophasic, or biphasic, with an early phase just after the onset of hypoxia/ischemia and a later one after several days. The degree of the altered permeability depends on the type, degree, and duration of occlusion [13].

The presence of comorbidities, such as hypertension and hyperglycemia, can induce anatomical and functional changes to the brain vasculature and often exacerbate BBB disruption after ischemia.

Currently, therapies for acute ischemic stroke are mostly based on tissue plasminogen activator-mediated thrombolysis, even if they are not always applicable [28]. 

### 3.2. Neurodegenerative Diseases

Alzheimer’s disease is one of the most common dementia disorders and is associated with cognitive decline and memory loss. AD is characterized by the presence of insoluble plaques of amyloid beta protein (Aβ) and neurofibrillary tangles constituted by hyperphosphorylated intraneuronal deposits of microtubule-associated protein tau (τ), which lead to neuronal cell death and loss of synapse [29]. This peptide aggregation is due to dysfunctional mitochondrial production of reactive oxygen species and dyshomeostasis of metals from oxidative stress [30]. Many studies have highlighted an increased extravasation of plasma proteins in AD brains, suggesting dysfunctional BBB properties. This dysfunction is probably caused by Aβ and τ accumulation in the perivascular areas and includes increased BBB permeability, microbleeds, reduced TJs’ expression, impaired transporter expression, accumulation of blood-derived products, and degeneration of PCs and ECs. Thus, toxic molecules, cells, and pathogens can enter the brain and trigger the inflammatory response, leading to disease progression and eventually causing cerebral amyloid angiopathy [31].

Parkinson’s disease is another common neurodegenerative disease affecting 2–3% of the population over 65 years old with motor dysfunctions, including tremor, rigidity, akinesia or bradykinesia, and postural instability. It is characterized by neural loss in the substantia nigra causing striatal dopamine deficiency, and Lewy is made of misfolded α-synuclein, neurofilaments, and ubiquitin in dopaminergic neurons and glial cells [32]. 

In PD, there is a BBB disruption detected by the increase in albumin level and immunoglobulin G in CSF, erythrocytes, hemoglobin, and fibrin extravasation in the striatum, and the reduction in ZO-1 and occludin [13]. There is no cure for PD, but some treatments could prevent the progression of the disease, such as the administration of DOPA agonists, DOPA precursors (or L-DOPA), amantadine, and anticholinergics [33]. 

Huntington’s disease is caused by an autosomal-dominant mutation: an expanded trinucleotide repetition of the CAG sequence in the gene HTT5 on chromosome 4 due to the abnormal pathogenic multifunctional protein huntingtin. It results in a progressive loss of neural function, resulting in movement, cognitive, and psychiatric problems, influenced by epigenetic, oxidative stress, metabolic, and nutritional factors. Post-mortem magnetic resonance (MR) studies showed increased BBB permeability, fibrin deposition, and a reduction in occludin and claudin-5 expression [13]. Currently, there is no effective therapy for the treatment or the reduction in HD progression [33]. 

Multiple sclerosis (MS) is the most common non-traumatic disabling disease affecting young adults, with an increasing incidence worldwide.

The onset of MS is associated with peripheral immune activation followed by CNS immune aggression, which causes demyelination and axonal loss, leading to neurodegeneration and irreversible neurological impairment. The most important pathological hallmarks of MS are BBB disruption, changes in the BBB endothelium, and lymphocyte trafficking [34]. It is generally considered a two-stage disease, characterized by early inflammation responsible for relapsing–remitting disease and then delayed neurodegeneration causing non-relapsing progression [35]. 

Prion diseases, or transmissible spongiform encephalopathies, are rare and fatal neurodegenerative disorders, including Creutzfeldt–Jakob disease, Gerstmann–Sträussler–Scheinker syndrome, fatal familial insomnia, and kuru in humans. Prions are nucleic acid-free structures, mainly composed of scrapie prion protein, a misfolded isoform of the host-derived cellular prion protein, and this transition is a key event for prion infection and propagation. The etiology of prion diseases can be sporadic, inherited, or caused by iatrogenic or dietary prion assumptions and lead to spongiform changes, neuronal loss, and neuroinflammatory responses [36]. 

### 3.3. Brain Tumors

Brain tumors are a heterogeneous group of both benign and malignant cancers in the brain parenchyma and surrounding tissues. They have relevant morbidity and mortality in both adults and children, often generating severe disabilities [37]. They can be classified as primary, when they arise from the glia and are usually called gliomas, or metastatic, when they originate from systemic cancers and further form metastasis in the brain parenchyma.

The most common ones are astrocytomas, oligodendrogliomas, and oligoastrocytomas. Astrocytoma arises from the ACs and is classified from grade I to grade IV, depending on histological findings. Grades I and II are low-grade, grade III is high-grade or anaplastic astrocytoma, and grade IV is also called glioblastoma multiforme (GBM) or malignant astrocytic glioma and is the most aggressive type. Oligodendrogliomas originate from oligodendrocytes or a glial precursor cell, while oligoastrocytomas have a mixed appearance of glial cell origin, astrocytoma, and oligodendroglioma. 

The major hurdles in effective gliomas’ treatments are related to the complex anatomy of the brain, to the difficulties in identifying tumor burdens, to chemoresistance, and to the concrete possibility of reaching the tumor sites in a therapeutic dose, avoiding overtreatments, and reducing the incidence and severity of adverse effects [38].

### 3.4. Infectious Diseases

Infections of the brain are caused by pathogens entering the BBB that normally prevent microbial invasions. Bacteria, viruses, fungi, and parasites can cause infections in the meningeal or parenchymal compartments, leading to meningitis or encephalitis, respectively [39,40,41]. 

Bacterial infections could be limited to localized focal infections, such as brain abscesses, or spread to meningoencephalitis. Bacteria could reach the brain from the upper airways, through the bloodstream from another primary site, or from a direct inoculation due to an injury or surgery. The most typical bacteria affecting the human brain are *Streptococcus pneumoniae*, *Neisseria meningitidis*, *Hemophilus influenzae*, and *Listeria monocytogenes*, while *Escherichia coli* and group B *Streptococcus* can affect neonates [42]. 

Viral meningitis and encephalitis are the most frequent brain infections, mostly caused by enteroviruses, parechoviruses, herpes simplex, varicella-zoster, Epstein–Barr virus, rabies, human immunodeficiency virus (HIV), measles, and COVID-19. These viral infections could potentially lead to neurological disorders [43,44]. Viruses can affect the brain in three different ways: by a direct invasion, causing encephalitis, inflammation, or necrosis, leading to permanent disability or death, as a result of a viral infection elsewhere in the body, from where inflammatory cytokines reach and cross the BBB, or this infection in another site may damage the brain through a long-range action through other mechanisms [45].

Fungal brain infections are usually opportunistic in immunocompromised patients, even though immunocompetent people with predisposing environmental and iatrogenic factors can be possible hosts. These infections originate from the direct inoculation of fungal spores, coming from yeasts, molds, and dimorphic fungi, after trauma or surgery. The most common infection is cryptococcal meningoencephalitis; candidiasis is a typical nosocomial infection; aspergillosis and mucormycosis, although rare, are devastating in immunosuppressed patients; and cerebral phaeohyphomycosis strike immunocompetent people [46,47].

Brain parasitic diseases are a huge issue, especially in low- and middle-income countries. The symptoms are very unspecific, such as meningitis, encephalitis, ventriculitis, myelitis, or brain abscess, with fever and headaches. Nematode infections cause eosinophilic meningoencephalitis, *Taenia solium* neurocysticercosis, which leads to epileptic seizures, some protozoan species, and free-living amoebae [47,48].

## 4. Drug Delivery across the BBB

At this point in the discussion, it should be clear that the BBB plays a key role in determining the success or failure of a therapy for any brain pathology. The role of this barrier is crucial because it must allow a specific drug to reach the exact site to be treated in the brain in the right dose and because, at the same time, it must prevent drugs used to treat pathologies in other areas of the human body from causing neurological damage.

Below is a list and description of all the ways in which it is possible to carry out therapies in the brain across the BBB by bypassing it, temporarily disrupting it, or by means of ligand conjugation.

### 4.1. Bypassing the BBB

#### 4.1.1. Intracerebroventricular

The intracerebroventricular (ICV) administration route consists of the direct injection of the drug into the CSF of the lateral cerebral ventricle after the penetration of the skull, using a catheter with an implantable reservoir or a pump. The pump is the most used since it guarantees a more continuous and elevated concentration of drug in the CSF. This method of administration allows a reduction in systemic side effects and avoids drug metabolism in blood serum and opsonization [49]. However, ICV administration has some significant drawbacks and risks. The CSF is turned over every 4–5 h via bulk flow and absorbed into the bloodstream; conversely, the ICV-infused drug can penetrate the brain by diffusion. The rate of CSF bulk flow is orders of magnitude greater than diffusion, so drugs often exit the ventricles faster than they can diffuse into the brain. In addition, the process is invasive and is often associated with other risks. For instance, catheter placement risks include hemorrhage, postoperative infection, and mispositioning. Some drugs could cause seizures and chemical arachnoiditis that could turn into leukoencephalopathy, but infections remain an important adverse effect that occurs either during the insertion of the device or for improper aseptic reservoir access [50,51,52].

#### 4.1.2. Intracerebral/Intraparenchymal

Intracerebral or intraparenchymal (IC/IP) administration is the most direct method since it delivers the drugs directly to the brain site through an implant or injection, and it spreads with a passive diffusion mechanism [53]. As with ICV, the process is very slow, and the drug can diffuse only up to 2 mm from the site of injection.

#### 4.1.3. Convection-Enhanced Delivery

Convection-enhanced delivery (CED) is a stereotactically guided drug delivery method in which the drug is delivered directly into targeted brain parenchymal cells. After a minimally invasive surgical exposure of the brain, one or more small catheters or micro-infusion pumps are placed into the parenchyma, allowing the delivery of drugs and ensuring a sustained therapeutic concentration [54]. CED has demonstrated potential utility in treating brain malignancies, but there are two main problems: the first is the high-flow-rate infusion for a uniform distribution across a large volume, and the second is the use of a large cannula to achieve this flux. The high flow rate induces damage to tissues at the infusion site and induces backflow along the insertion tract. At the same time, the large cannula can lead to inflammation, tissue damage, and scarring around the device [55].

#### 4.1.4. Implants

Polymeric implants and interstitial wafers are often used, for example, for glioblastoma’s treatment. However, they have poor drug penetration beyond the resection cavity, drug dosage is limited by the implant size, and they are associated with high intracranial pressures and local toxicity, causing infections and brain trauma [54,56,57].

#### 4.1.5. Intranasal Delivery

Intranasal delivery is a non-invasive approach for the delivery of therapeutics to the brain across the olfactory mucosa and along the connective tissue around the olfactory nerve bundle or axons of olfactory neurons, thus bypassing the BBB. This approach, guaranteeing a nose-to-brain direct delivery, enhances drug targeting and bioavailability with a faster brain delivery and, avoiding the metabolism of the liver, decreases drug accumulation in non-targeting tissues, minimizing side effects [58].

Once the intranasal-administered molecules reach the origins of the olfactory and trigeminal nerves in the cerebrum and pons, respectively, they are dispersed throughout the brain following different mechanisms: intracellular and extracellular. In the intracellular one, the molecule is internalized by an olfactory neuron, transported by an endocytic vesicle to the neuron’s projection site, and then exocytosed. Conversely, in the extracellular pathway, the molecule crosses the nasal epithelium, reaching the lamina propria, where the neurons are located, and it is transported externally along the neuronal axon by bulk flow processes [59,60]. 

Besides the advantages of this administration method, it also has some drawbacks due to the poor permeability of drugs from the nasal mucosa, their enzymatic degradation and mucociliary clearance, the low retention time, and nasomucosal toxicity. The clinical application of intranasal delivery is limited by the necessary high and frequent doses since each human nostril has an administration volume <200 μL and at most, 1% of the drug reaches the brain, moreover, strongly irritating the nasal mucosa [61]. In addition, the formulations have a short residence time in the nose (15–30 min), which confines the drug adsorption. Furthermore, the enzymes of the nasal cavities can enzymatically metabolize many sensitive drugs, and the formulations must have pH values and viscosities compatible with those of the nasal mucosa, such as not inducing irritation or inflammation of the nasal epithelium [62]. 

The most commonly used nanocarriers for intranasal delivery are SLNs and NLCs for their improved nasal retention, biocompatibility, drug solubility and permeability, reduced mucociliary clearance, and drug enzymatic degradation. 

### 4.2. Temporary Disruption of the BBB

The temporary disruption of the BBB is the most commonly used method to deliver drugs from the bloodstream to the CNS. 

Besides the pathological degradation of the BBB, where a therapeutic nanocarrier administered intravenously could, in theory, directly accumulate in these diseased regions, temporary induced BBB permeability could be achieved with some strategies, such as the administration of chemical agents, ultrasounds (US), and magnetic fields [63]. 

#### 4.2.1. Osmotic Disruption 

The temporary osmotic BBB disruption is based on the high osmotic pressure induced by the administration of chemical substances such as mannitol, fructose, milk amide, urea, and glycerol. The injection of hyperosmolar agents at a flow rate sufficient to allow a complete filling of the vessel without producing significant reflux in the common carotid artery leads to the reversible dehydration of brain ECs and subsequent disruption of the TJs. This method intervenes in the overcoming of the sink effect, which is the accumulation of the chemotherapeutic drug in tumor necrotic areas, seizing them from the periphery of the tumor, such as the highly proliferative tumor edges with neoplastic cells. In fact, ensuring a more uniform delivery to the entire CNS vascular territory, including tumor edges, osmotic disruption (OD) provides longer tumor cell exposure to higher concentrations of the drugs [64]. However, BBB’s OD temporarily increases the fluid influx, potentially leading to a transient cerebral edema and the entrance of not only molecular components, which can cause neuropathological changes, neurological toxicity, aphasia, and hemiparesis, but also toxic and harmful agents, possibly resulting in a change in the normal functions of the CNS [58,64].

#### 4.2.2. Ultrasound Disruption

The transient disruption of the BBB using high-intensity focused US is based on the combination of US, that can pass through the skull and converge at a specific focal point inside the brain and intravenously injected microbubbles (MBs). These MBs, excited by the rarefactions and compressions provided by US, start to oscillate, exerting a mechanical stress on the cells, leading to the opening of TJs. This BBB disruption effect lasts usually for 4–6 h, but it can vary according to the patient, the intensity of US, and the size and concentration of the MBs [58,64]. In addition, these US favor the active transport of molecules across the BBB, for example, by enhancing the delivery through vesicles and carrier proteins or modulating mechanosensitive ion channels, they can cause convective flux in the tumor interstitial space, and they can remodel brain vasculature and stimulate the development of new neurons [65,66,67]. This approach is currently on clinical trials for gliomas (NCT03322813, NCT02343991, NCT03616860, and NCT03551249) [68,69,70], recurrent GBM (NCT02253212, NCT03626896, and NCT03712293) [71,72,73], amyotrophic lateral sclerosis (NCT03321487) [74], PD (NCT03608553 and NCT04370665) [75], and AD (NCT02986932, NCT03671889, NCT03739905, and NCT04118764) [76,77,78]. Although undoubtedly advantageous, especially for big molecules (500–2000 kDa), BBB US disruption also provides inherent risks, such as large volumetric oscillations of the MBs and, potentially, their collapse, which can generate extra mechanical stresses on the capillaries in the form of micro-jets that can damage the surrounding capillary and brain parenchyma [64,79].

#### 4.2.3. Optical Disruption

The optical disruption of BBB consists in the illumination of a brain region, inducing the internalization of junction molecules and locally opening the BBB for up to 48 h. This optical disruption can be obtained in two different ways: using photodynamic or laser interstitial thermal therapy (LITT) [80]. In the first way, the light activates some photosensitizers (such as fluorescent dyes), which generate reactive oxygen species, inducing changes in junction morphology and increasing BBB permeability with high spatiotemporal selectivity [81]. LITT uses laser energy to generate heat in the target tissue, and it can temporally increase the BBB permeability, enhancing the secretion of heat shock proteins and nitric oxide [80]. LITT, applied to enhance BBB permeability, has undergone some clinical trials, such as NCT01851733 in combination with doxorubicin [82] or NCT02311582 with pembrolizumab [83]. The optical disruption of the BBB requests the exposition of the brain by creating an optical window in the skull.

#### 4.2.4. Electrical Disruption

The electrical disruption of the BBB can be achieved either by transcranial stimulation applying electrodes to the skull or by using penetrating electrodes to generate pulsed electric fields (PEF) to provoke and electroporation (EP) of the BBB. In transcranial stimulation, there is increased permeability, probably due to the increased convection through the gaps between TJs. When PEFs are applied to cells or tissue, they change the innate electrical potential across the cell membranes. The destabilization of membrane potential creates nanoscale aqueous pores in the lipid bilayers, resulting in an increased membrane’s permeability, termed electroporation. After the EP, if membranes reseal, it is called a reversible EP, while if it leads to cell death, it is an irreversible EP [84]. Electrical stimulation has shown cognitive and therapeutic effects, but its safety, efficacy, and the potential risk of increased exposure to toxins and pathogens have not been fully evaluated. In addition, EP probes are highly invasive, and if they must be inserted in deep brain regions, they could cause long-term damage [80].

#### 4.2.5. Radiation Therapy Disruption

Since ECs and oligodendrocytes are radiation-responsive, ionizing radiation can be used in a controlled and targeted way to selectively damage BBB tissues, increasing their permeability. High doses of radiation can favor BBB permeability through TJ modifications, cell density reduction, and the formation of actin stress fibers. Few clinical trials, such as NCT02974803, have been conducted using this method despite showing promising results; the optimal radiation dose and therapeutic window are still not determined, and the side effects remain serious [85]. 

### 4.3. Ligands Conjugation for Active Brain Targeting

Besides the just-described methods to bypass or temporarily disrupt the BBB for drug delivery to the brain, many nanotechnological solutions have been designed to enhance BBB penetration [86]. In the following paragraph, some of the most commonly used functionalization approaches have been reported. The delivery of drugs to the brain occurs through different mechanisms, according to molecules’ physicochemical properties, such as adsorptive-mediated transcytosis, receptor-mediated transcytosis, transporter-mediated transcytosis (TMT), and cell-mediated transcytosis [4,87]. 

#### 4.3.1. Adsorptive-Mediated Transcytosis

##### Cardiolipin

Cardiolipin is a component of the mitochondrial membrane and is necessary for numerous enzymatic activities for mitochondrial energy metabolism. Since cardiolipin is positively charged, it can cross the BBB through AMT. Interestingly, in the case of AD, nanocarriers functionalized with cardiolipin cannot by themselves decrease Aβ fibrils in the brain, but they reveal a high affinity for these fibrils, opening new perspectives for the generation of new vehicles for imaging and new therapeutic agents [87].

##### Heparin

Heparin is a polyanionic polysaccharide of the glycosaminoglycans family widely used in nanomedicine in the oncological, coagulation, tissue engineering, and drug delivery fields. It demonstrated an innate ability to compete with Aβ peptides in binding to proteoglycans, properties particularly interesting for AD therapy [87]. 

##### Cell-Penetrating Peptides 

Cell-penetrating peptides (CPPs) are small cationic or amphipathic peptides that can be translocated across cell membranes, delivering the associated compounds inside cells without compromising their properties, exploiting the presence of peptide sequences called protein transduction domains. They also have a positive charge, which can favor electrostatic interactions with membranes. 

An example of CPP is poly-l-Arginine, which is a synthetic cationic peptide constituted by eight or more arginine residues and is one of the most widely used peptides in drug delivery. Another CPP is penetratin, which enhances internalization across epithelial cells in a two-step mechanism: penetratin binds cell-surface lipids through electrostatic interactions and is translocated via tryptophan-induced destabilization [88]. Other CPPs are penetratin, SynB, HIV-1 trans-activator of transcription (TAT) protein, and octa-arginine (R8) [4].

#### 4.3.2. Receptor-Mediated Transcytosis

##### Transferrin Receptor

Transferrin (Tf) receptors (TfR) are transmembrane glycoproteins constituted of two subunits of 90 kDa linked by a disulfide bridge, and each of them can bind one molecule of transferrin [88]. TfR can be exploited for brain delivery since it is overexpressed on brain capillary endothelial cells, but it must be considered that TfR is also expressed on other cells, such as hepatocytes and monocytes, besides BBB, that the high concentration of endogenous serum transferrin typically saturates all the receptors, and that it can lead to an overdose of iron transport into the brain [89,90]. To overcome these limitations, NPs can be conjugated with the transferrin monoclonal antibody (OX-26), since they bind to a different site than the transferrin protein, interfering less with endogenous transferrin [90]. Another alternative is T7 (HAIYPRH), which is a heptapeptide that can bind to TfR with high affinity without any competitive inhibition with endogenous Tf since they bind to different sites of TfR [88].

##### Lactoferrin Receptor

The lactoferrin (Lf) receptor (LfR), a single-chain cationic-iron-binding glycoprotein of the TfR family, is constituted by a homodimer and has anti-inflammatory, antimicrobial, and immunomodulatory functions. The LfR has two binding sites, a high-affinity and a low-affinity one, and their sizes and features change depending on the cell types, opening the possibility of targeting a particular LfR [58,91]. Tf and Lf have similar characteristics, but lactoferrin has a lower plasma concentration and a unidirectional brain uptake mechanism [88]. In addition, LfRs are overexpressed on the BBB in several neurological conditions, such as AD, PD, and HD [92].

##### Lipoproteins Receptor

Low-density lipoprotein (LDL) receptor (LDLR) is the removal of highly atherogenic LDL from the circulation; in particular, LDLR, LDLR-related protein (LRP) 1, and very low-density lipoprotein receptor are overexpressed on brain ECs. Alipoproteins B and E (ApoB and ApoE) are soluble apolipoproteins that bind to the LDL receptor and, thus, can be exploited to cross the BBB. ApoE and apoB have demonstrated efficacy but have innate protein instability and compete with LDL [88]. An alternative to ApoE functionalization is the coating of NPs with Tween 80, since it induces the adsorption of ApoE present in the bloodstream’s systemic circulation. 

Angiopep-2 is a 19 amino acid peptide derived from the Kunitz domains of aprotinin and other human proteins, which are ligands for LRP1 and LRP2, and it can induce the crossing of the BBB through the recognition of LDL receptors. Angiopep-2. It has a high transcytosis capacity, bypassing the P-glycoprotein efflux pump. Functionalization with angiopep-2 can increase the concentration of the nanocarrier in the brain tumor site, probably attracted by the acidic tumor microenvironment.

##### Nicotinic Acetylcholine Receptors 

The nicotinic acetylcholine receptor binds the neurotransmitter acetylcholine and is widely expressed in not only the brain in pre- and postsynaptic sites of neurons but also the BBB. They are a very promising tool for drug delivery since they allow the passage of the BBB but also target neuronal cells. This receptor could be exploited by the rabies virus glycoprotein (RVG) 29 peptide, a 29 amino acid fragment from the rabies virus glycoprotein.

#### 4.3.3. Transporter-Mediated Transport

##### Glutathione 

Glutathione (GSH) is a hydrophilic tripeptide known as an antioxidant useful to maintain cellular redox homeostasis and suppress oxidative stresses. GSH has been evaluated for brain drug delivery by exploiting the TMT via GSH transporters.

##### Acetycholine 

Acetycholine is an essential neurotransmitter that requires choline to be synthesized and transported to the brain via choline transporters.

##### Glucose

Glucose is the essential fuel of the brain that must be transported from the bloodstream to the brain by dedicated transmembrane proteins, the glucose transporters (GLUT), since neurons are unable to synthesize or store glucose. GLUT is expressed in the BBB to mediate the uptake of the metabolites but is overexpressed in brain cancer cells, making them attractive for glioma treatment. GLUT1, which transports glucose from the blood to the extracellular spaces, and GLUT3, which transports glucose from the extracellular space to neurons, are the main transporters in the human brain and are present in approximately equal amounts. Although there are some concerns regarding the number of these transporters in some pathologies, such as AD and hyperglycemia, GLUT can be effectively used for brain targeting [88].

#### 4.3.4. RGD Peptides

RGD (arginine–glycine–aspartic acid) tripeptide has been widely studied for drug delivery applications for its affinity with the ECM proteins, fibronectin and vitronectin, and integrin αvβ3/αvβ5. Cyclic forms of RGD such as c(RGDyK), c(RGDfC), c(RGDfK), RGD4C (KACDCRGDCFCG), and iRGD (CRGDK/RGPD/EC) have higher stability in biological environments. Moreover, iRGD can also act as a tumor-penetrating peptide, binding to the neuropilin-1 (NRP-1) receptors on tumor cells [93].

#### 4.3.5. Antibodies

Vascular endothelial growth factor (VEGF) and its type II receptor, VEGFR2, are highly expressed in brain tumors, playing a key role in angiogenesis and metastasis. Thus, gliomas’ and GBMs’ antiangiogenic therapies aim to inhibit angiogenesis by using anti-VEGF monoclonal antibodies (mAbs) like bevacizumab or avoid VEGF binding to the receptor by blocking VEGFR2 with an mAb such as ramucirumab [93]. 

#### 4.3.6. Aptamers

Aptamers are short, single-stranded sequences of DNA or RNA that can bind to their receptors with high affinity and specificity. If compared to antibodies, aptamers have higher stability, lower immunogenicity, a small size (5–30 kDa), and a simple synthesis and modification procedure [93].

#### 4.3.7. Polyethylene Glycol 

Coating lipid-based nanocarriers (NCs) with polyethylene glycol (PEG) is a widely known method to prolong the NCs circulation time in plasma and their chemical stability. In the case of brain delivery, PEG confers a BBB crossing ability depending on the chain length; longer chains demonstrated better efficiencies in a time-dependent manner [89].

## 5. Lipid-Based Nanocarriers

The major hurdle in drug delivery to the brain is the presence of the BBB and enzymes, which strictly select the substances that could enter the brain. There are several factors that could be controlled to drive the entry of compounds into the brain, such as binding the drug to a transporter, opening and closing ion channels, lipophilicity, enzymatic degradation of drugs, functional groups, and charged residues of the molecules. 

Furthermore, after penetration inside the brain, the drug faces other hurdles, such as inactivation by catabolic enzymes, drug resistance, and affinity towards multidrug ABC transporters, making the drug less bioavailable to the target site. Some studies estimate that almost 98% of small-molecule drugs and mostly all the large-molecule ones are excluded from entering the BBB [54]. Thus, alternative strategies are required to enable the treatment of CNS disease. The growing number of patients with CNS diseases urgently requires the development of new and non-invasive drug delivery methods as alternatives to traditional surgery, radiotherapy, and chemotherapy. In this vision, nanotechnologies are emerging as a good alternative to classical treatments for their ability to directly alleviate oxidative stress and inflammation, overcome the BBB, deliver therapeutics in a targeted manner to the site of disease, enhance the dose efficacy, control the release profiles, and avoid side effects.

Thanks to the many advances in nanotechnology, nanomedicine has a wide array of organic, inorganic, or NCs for therapeutic applications [94,95]. Among the organic NCs, those that seem to be most used for brain drug delivery are the lipid-based ones, that is, liposomes, SLNs, NEs, NLCs, niosomes, proniosomes, cubosomes, EVs, cell membrane-derived nanocarriers, and organic nanocarriers with high lipophilicity and ability to cross the BBB through passive diffusion [86,96].

### 5.1. Liposomes

**Figure 3:**
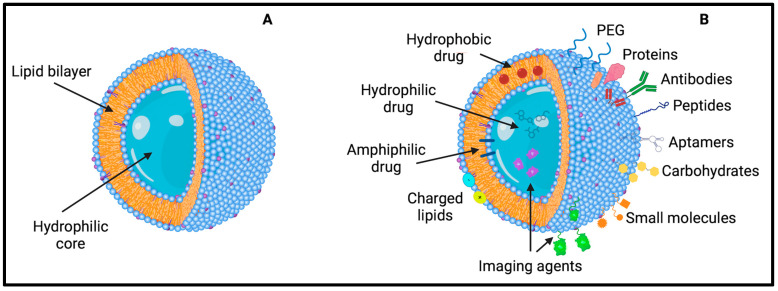
Representation of (**A**) liposomes’ structure made of a lipid bilayer encapsulating an aqueous core and (**B**) their possible load and functionalization.

Liposomes are synthetic or natural self-assembled lipid bilayers deeply studied since 1960 for their applicability as drug delivery vehicles thanks to their structural similarity to biologic membranes. They are biocompatible, non-toxic, and biodegradable, making them suitable for drug delivery, preventing drugs’ degradation and immune responses [97]. They are composed of a hydrophobic bilayer and a hollow aqueous core, allowing the encapsulation and delivery of hydrophilic, hydrophobic, and amphiphilic molecules, such as proteins, peptides, nucleic acids, small molecules, and drugs (Figure 3). 

Liposomes are widely used to overcome some limitations of bare therapeutics. Some liposomes have already been approved by the FDA for the treatment of many brain pathologies or are undergoing some clinical trials, as follows:

- AmBisome^®^ for the treatment of cryptococcal meningitis, composed of amphotericin B encapsulated in a lipid bilayer of hydrogenated soy phosphatidylcholine (HSPC), 1,2-distearoyl-sn-glycero-3-phosphoglycerol (DSPG), and cholesterol (Chol);
- Abelcet^®^ for the treatment of cryptococcal meningitis, composed of amphotericin B encapsulated in a liposome made of 1,2-dimyristoyl-sn-glycero-3-phosphocholine (DMPC), and 1,2-dimyristoyl-sn-glycero-3-phosphoglycerol (DMPG);
- Daunoxome^®^, composed of distearoylphosphatidylcholine (DSPC) and Chol liposomes carrying daunorubicin for the treatment of pediatric brain tumors;
- Depocyt^®^, cytarabine encapsulated in Chol, triolein, 1,2-dioleoyl-sn-glycero-3-phosphocholine (DOPC), and 1,2-dipalmitoyl-sn-glycero-3-phosphoglycerol (DPPG) liposomes for the treatment of lymphomatous meningitis;
- Doxil^®^/Caelyx^®^ also proposed for the treatment of GBM and pediatric brain tumors by encapsulating doxorubicin in HSPC, Chol, and 1,2-distearoyl-sn-glycero-3-phosphoethanolamine poly(ethylene glycol) 2000 (DSPE-PEG2000) liposomes;
- Myocet^®^ liposome composed of egg phosphatidylcholine (EPC) and Chol-encapsulating doxorubin were also proposed for the GBM [8].

Some already-approved liposomal formulations have been repurposed, such as Depocyt^®^ for the treatment of patients with recurrent GBM [98], liposomal amphotericin B for CNS infections with azole-resistant *Aspergillus* [99], or recurrent *Candida albicans* meningitis [100], and liposomal cytarabine for pediatric malignant brain tumors [101], administering them through ICV or IC.

Besides all the benefits, liposomes also display some limitations. That is, fast clearance and degradation and stability issues after prolonged storage times [89]. To face these hurdles, different formulations and strategies have been studied to enhance drug delivery across the BBB. Besides the lipophilic features of liposomes, they are too large to simply diffuse across or between cells, but they must exploit transport systems such as AMT, RMT, and CMT. To cross the BBB via the abovementioned routes, liposomes can be functionalized to enhance their blood circulation time or to improve their targeting ability in the CNS [85,89]. 

The first modification is the formulation of cationic liposomes to use the AMT. They display a positive surface charge to favor the electrostatic interaction with negatively charged glycocalyx at the luminal BBB membrane. Moreover, the liposomal positive charge enhances the adsorption of polyanions, such as DNA and RNA. The brain uptake of liposomes strongly depends also on their adhesion force to BBB, which must overcome the hydrodynamic force of cerebral blood flow, which can be affected by the administration routes and pathologies. For instance, a large cationic liposome (~200 nm) is preferred when the wall shear rate is low, such as in the case of transient cerebral hypoperfusion due to intra-arterial injection. On the contrary, a smaller one is better when the blood flow is faster and the hemodynamic stress is high. Thus, liposomes’ particle size must be optimized, considering the hemodynamic stress factors [102].

To avoid the problem of fast clearance, PEG can be covalently conjugated to liposomes to enhance their stability and prolong their circulation half-life. PEGylation inhibits liposomes’ clearance by the mononuclear phagocytic cells in the liver and spleen, preventing their opsonization. However, PEG chains can hinder the uptake of liposomes by target cells by impeding the binding of surface-targeting ligands with the matching cell surface receptor [103,104]. 

Furthermore, liposomes can be functionalized by targeting biological moieties, such as proteins, antibodies, carbohydrates, aptamers, and polypeptide sequences, through covalent or non-covalent bonds. Covalent bonding includes thioether, hydrazone, carboxamide, amide, and disulfide bonds, while non-covalent or physical bonding relies on attractive forces such as electrostatic interactions, hydrogen bonding, and van der Waals forces. However, non-covalent bonding has some problems in the control of the orientation of the ligands, hindering the liposome’s stability and activity, and of the environmental conditions required since changes in ionic strength, pH, or the isoelectric point of the ligand can lead to the detachment of the ligand from the surface [104].

Some liposomes are currently undergoing clinical trials, as follows:

NCT05034497, NCT01906385, and NCT05460507: Rhenium-186-NanoLiposome are administered through CED [105,106] or ICV injection to allow localized GBM therapy.

- NCT05768919: liposomal curcumin is associated with radiotherapy and temozolomide for patients with newly diagnosed high-grade gliomas (HGG).
- NCT00944801: pegylated liposomal doxorubicine and temozolomide in addition to radiotherapy in newly diagnosed GBM [107].
- NCT04573140: RNA-lipid particle vaccines are used for the therapy of newly diagnosed pediatric HGG and GBM [108].
- NCT00019630 and NCT00465673: liposomal doxorubicin HCl for the pediatric treatment of refractory brain tumors or brain metastases from breast cancer.
- NCT00992602: IC injection of liposomal cytarabine combined with methotrexate for breast cancer brain metastasis.
- NCT01386580 and NCT01818713: a GSH-functionalized pegylated liposome loaded with doxorubicin hydrochloride is administered in patients with HGG and leptomeningeal breast cancer metastasis [109].
- NCT04590664: a repurposing of the drug verteporfin for the treatment of recurrent high-grade EGFR-mutated GBM [110].
- NCT05864534: liposomal doxorubicin is administered in combination with a device with nine US emitters to disrupt the BBB and enhance drug penetration into the brain tumor.
- NCT01044966: ICV administration of liposomes encapsulating Ara-C (DepoCyt^®^) in patients with recurrent GBM.
- NCT00734682: liposomal irinotecan for recurrent HGG [111,112].
- NCT03086616 and NCT02022644: CED of irinotecan liposome with real-time imaging with gadolinium in children with diffuse intrinsic pontine glioma and adults with HGG [113].
- NCT01356290: oral thalidomide, fenofibrate, celecoxib, and alternating 21-day cycles of oral etoposide and cyclophosphamide, supplemented by intravenous bevacizumab and intraventricular therapy via an Ommaya reservoir consisting of alternating etoposide and liposomal cytarabine for children with medulloblastoma and ependydoma [114].
- NCT01222780: Marqibo^®^ (liposomal Vincristine) for children and adolescents with refractory tumors.
- NCT05496894: mitoxantrone hydrochloride is encapsulated in a liposomal formulation for the treatment of MS.
- NCT01039103: intravenous PEG-liposomal prednisolone sodium phosphate (Nanocort^®^) for the treatment of MS.
- NCT02686853: intrathecal administration of liposomal amphotericin B in cryptococcal meningitis in immunocompetent patients;
- NCT05453539: a novel liposomal device constituted by DSPE-DOTA-Gadolinium for contrast-enabled MR imaging of amyloid plaques for the diagnosis of AD.
- NCT04976127: liposomal talineuren for PD.

Otherwise, many liposomal formulations are still undergoing preclinical studies, and some examples are reported in Table 1.

### 5.2. Solid–Lipid NPs

Similar to liposomes, solid–lipid NPs are nanometric lipid-based constructs, but they have a solid hydrophobic lipid core, in which both hydrophilic and lipophilic drugs can be encapsulated. They were developed for the first time in the early 1990s by Müller et al. and found application, especially in the cosmetic field [156].

**Figure 4:**
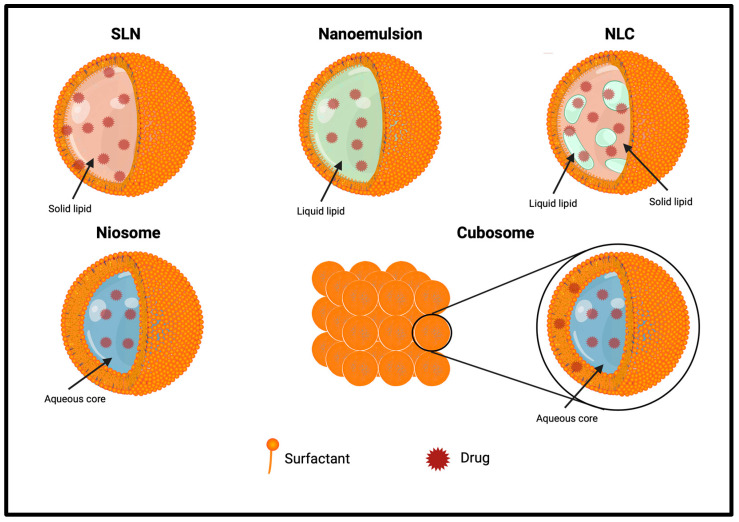
Structure of other synthetic lipid-based delivery systems.

The main feature of SLNs is that they contain solid lipids at room temperature (Figure 4). Its solid lipidic core, instead of an aqueous one, protects drugs from biochemical degradation. SLNs have excellent physicochemical stability that allows them to escape the reticuloendothelial system by bypassing liver and spleen filtration; they are physiological and biodegradable, with a high biocompatibility; and their fabrication is scalable, fast, and economic.

The solid lipid core allows SLNs’ storage for a long time in aqueous solutions, which is impossible with liposomes for the establishment of degradation phenomena.

However, SLNs also have some drawbacks, such as poor drug loading due to the limited space in the organized solid lipid core and the possible interaction of the drug with the lipid matrix, resulting in a failure of the formulation [157,158].

Some SLN formulations are undergoing promising preclinical studies, as listed in Table 2.

### 5.3. Other Synthetic Lipid Nanocarriers

Besides liposomes and SLNs, there are other lipid-based synthetic NCs, such as nanoemulsions, nanostructured lipid carriers, niosomes, proniosomes, and cubosomes, that are studied for brain delivery applications (Table 3, Figure 3).

#### 5.3.1. Nanoemulsions

Emulsions are biphasic liquid systems constituted by two phases, the internal one dispersed as a small droplet in the external or continuous one. Their main feature, making them extremely useful in the food, pharmaceutical, and cosmetic fields, is the possibility to mix non-polar and polar molecules. Among them, NEs are nanosized emulsions where surfactants are employed to lower the surface tension and act as a barrier to emulsion coalescence at the interface between the two phases. NEs are widely used in nanomedicine to solubilize hydrophobic drugs, reducing side effects [169].

#### 5.3.2. Nanostructured Lipid Carriers

NLCs are the second generation of SLNs, developed in 1999, and they are defined as nanometric (50–500 nm) colloidal drug delivery systems, containing a lipid mixture of both solid and liquid lipids in their core. Compared to SLNs, which have a solid lipid core in a highly organized fashion, NLCs contain liquid and solid lipid, forming an unorganized drug matrix. This unorganized nature allows the encapsulation of more drugs in the core and prevents crystallization and drug leakage during storage. They are biocompatible, non-toxic, and safe, with high stability and drug loading ability if compared to other lipid-based delivery tools [170].

#### 5.3.3. Niosomes and Proniosomes

Niosomes are nonionic surfactant vesicles, like liposomes, used to improve the solubility and stability of poorly soluble drugs. Proniosomes are water-soluble nonionic dehydrated powdered or gelated structured provesicles that can be immediately rehydrated before use, avoiding many issues related to aqueous vesicular dispersions [171]. They are constituted by a lipid compound, cholesterol, or L-α-soya phosphatidylcholine, and nonionic surfactants, such as spans, tweens, and Brij [172].

#### 5.3.4. Cubosomes

Cubosomes are composed of amphiphilic lipids and surfactants organized in a cubic nanostructure. The presence of liquid–crystal phases favors the dissolution of hydrosoluble peptides. They have some advantages, such as easy formulations, biocompatibility, prevention from degradation, and stability [33].

**Table 3:** Nanoemulsions, nanostructured lipid carriers, niosomes, proniosomes, and cubosomes for the treatment of brain diseases.

LNC	Composition	Drug	Surface Functionalization	Size (nm)	ZP (mV)	Disease	Administration Route	Reference
NE	Capmul MCM + Tween 80 + Transcutol P + propylene glycol	Quetiapine fumarate	-	144.0± 0.5	−8.1 ± 1.8	Brain delivery	Intranasal	[173]
	Capryol PGMC + Kolliphore^®^ RH40 + Transcutol^®^-P	Zolmitriptan	Chitosan	43.5 ± 1.9	+5.2 ± 0.9	Migraine	Intranasal	[174]
	Isopropyl myristate + Capryol + Cremophor EL + Labrasol	Huperzine A	Lf	15.2 ± 0.7	−4.5 ± 1.0	AD	Intranasal	[175]
	oleic acid + α-tocopherol + Span 8 + olive oil + Tween 80	Indinavir		112 ± 4	−33 ± 3	HIV	Intravenous	[176]
NLCs	Precirol ATO 5+ Capmul MCM + Tween 80 + Span 20	Carbamazepine	-	132.8	−29 ± 6	Epilepsy	Intranasal inside gel	[177]
	Compritol + Sweet almond oil + L-PC + gelucire 44/14	Flibanserin	-	115	-	Brain delivery	Intranasal inside gel	[178]
	Precirol ATO 5 + Lauroglycol 90 + Tween 80	Escitalopram and paroxetine	-	165 ± 2	+11.2 ± 0.4	Depression	Intravenous and intranasal	[179]
	Cetyl palmitate + oleic acid + Tween 80 + Polaxomer 188	Sesamol	-	92 ± 6	−27.9 ± 0.6	Ischemic stroke	Intravenous	[180]
	Compritol + Labrafil + Tween 80 + lauroglycol	Almotriptan malate	Chitosan	254.9 ± 1.9	+34.1 ± 0.1	Migraine	Intranasal	[181]
	Glyceryl monostearate + oleic acid + Tween 80 + pluronic F127	Lorazepam		72 ± 5	−20 ± 3	Epilepsy	Intranasal	[182]
	Palmityl palmitate + Miglyol^®^ + sphingosylphosphorylcholine + Solutol HS15^®^ + DSPE-PEG2000	Nimodipine	Lf	170 ± 14	−15.9 ± 1.1	Ischemic stroke	Intravenous	[183]
	PC + chol oleate + glycerol trioleate + S100-COOH	Curcumin		103.8 ± 0.6	−5.8 ± 0.7	AD	IC	[184]
Niosomes	Span60 + Chol	Bromocriptine mesylate	-	180 ± 5	−14.2 ± 1.8	Brain delivery	Intranasal	[185]
	DOTMA + lycopene + polysorbate 60	pCMS-EGFP plasmid	-	119 ± 3	+23 ± 2	Brain delivery	IC	[186]
	Chol + Tween60	Thymoquinone	-	78	−5	Ischemic stroke	Intravenous	[187]
	SUR II + Chol + PEG2000	Pramipexole	-	103 ± 0.4	−13.8 ± 0.2	PD	Intraperitoneal	[120]
	Tween60 + Chol	Oleuropein	-	79.37 ± 0.12	+1.38 ± 0.07	Metastatic brain tumors	Intravenous	[188]
	Span 60 + Solulan C24	Albumin	Glucopyranose and alanine	94 ± 10	−3.8 ± 1.0	Brain delivery	Intravenous	[189]
	Span + Chol	Olanzapine	Chitosan	250 ± 5	-	Schizophrenia	Intranasal	[190]
	Span 60 + Chol	Lacosamide		194	+36	Epilepsy	Intravenous	[191]
	Dicetyl phosphate + Chol + Tween20	Pentamidine		118 ± 2	−26.7 ± 0.7	Brain delivery	Intranasal	[192]
Cubosomes	Phytantriol + Tween80	-	-	170–250	-	Brain delivery	Intravenous	[193]
		Gold NPs	-	196 ± 3	-		Intravenous	[194]
	Selachyl alcohol + Tween80	Phenytoin	-	144 ± 4	-	Seizure	Intravenous	[195]
	Glycerol mono-oleate + poloxamer 407	Donepezil HCl	-	138–231	−40	AD	Intranasal	[196]
	Glycerol monooleate + Poloxamer 407 + Tween 80	Granisetron	-	267 ± 3	−27 ± 2	Chemotherapy-induced emesis	Intranasal	[197]
	Glyceryl monooleate + poloxamer 407 + ethanol + polyethylene glycol 200	Tizanidine hydrochloride	-	50.2	−6.4	Brin delivery	Intranasal	[198]
	Monoolein + Tween80	Paliperidone palmitate	Chitosan	306 ± 23	+42.4 ± 0.2	Schizophrenia	Intranasal	[199]
	Glyceryl monooleate + Pluronic 127	Gambogenic acid and PLHSpT	Angiopep-2	128.7 ± 1.0	>30	GBM	Intravenous	[200]
	Monoolein + amphiphilic polymer	Temozolomide or cisplatin		∼280	+18	GBM	Intravenous	[201]

DOTMA: 1,2-di-O-octadecenyl-3-trimethylammonium propane.

### 5.4. Extracellular Vesicles

**Figure 5:**
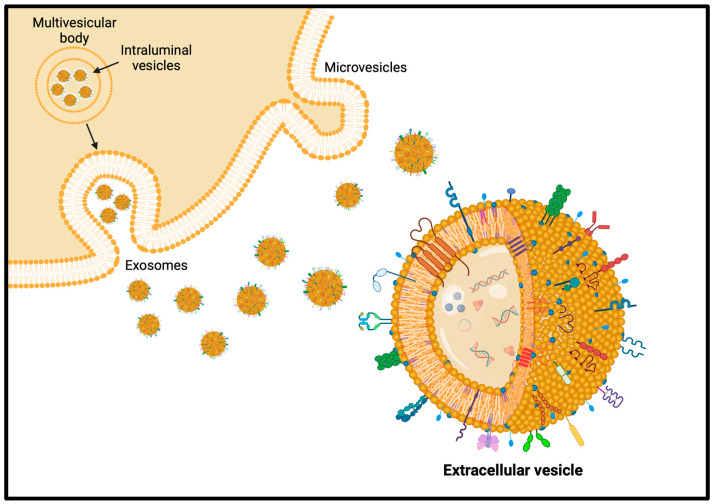
Representation of extracellular vesicles and their production and release mechanisms.

Extracellular vesicles (EVs) are phospholipid bilayer-delimited vesicles naturally produced by cells in both physiological and pathological conditions. Their membrane and cargo composition mirror the cell of origin and can modulate many physiological and pathological cellular processes, acting as effective intercellular communication mediators (Figure 5). In this way, EVs modulate immune reactions, tissue regeneration, tumor niche establishment, and tumor metastatization, triggering phenotypic changes in acceptor cells. This key role of EVs demonstrates their potential as vehicles for the delivery of therapeutic cargoes or as hybrid nanosized tools engineered ad hoc to regulate a physio-pathological condition or a disease progression. In addition to their delivery capabilities, EVs have intrinsic targeting abilities towards the parental or pathological tissue [202]. The increasing interest of researchers in EVs mainly relies on their potential diagnostic and therapeutic applications in many medical fields such as cancer, neurodegenerative, and immunological diseases, and many clinical trials involving different types of EVs, even from plants, are already on the go [203].

In addition to the just-described properties, EVs have the outstanding, and still not totally understood, ability to cross the BBB bidirectionally, influencing neurons or peripheral tissues through the bloodstream. The comprehension of this phenomenon becomes essential for the use of EVs as drug delivery vehicles in pharmacology and therapeutics. In the last decade, the effects of EVs have been evaluated in preclinical models of brain diseases such as AD, stroke, traumatic brain injury, and intracerebral hemorrhage. Similarly to other body compartments, EVs in the brain also play a key role in the communication between neurons, glia, and vascular cells, especially in the maintenance of homeostasis and the progression of pathologies. In the last decade, there has been a shift from cell-based therapeutics to EV-based ones, and in this regard, many studies have shown the potential of EVs as nanotherapeutics for brain pathologies. Native EVs have neuroprotective and regenerative effects, but they can also be engineered in terms of payload and surface functionalization to enhance their bioactivity and targeting [204]. 

Although EVs have unique properties to advance smart drug delivery systems in terms of pharmacokinetics, targeting, and safety against those of synthetic nanocarriers, clinical translation of these results is still challenging. The EVs’ intrinsic size heterogeneity, batch-to-batch differences, and the risks of the biogenesis procedure are higher than in synthetic nanocarriers. Moreover, effective and reproducible methods to load them with therapeutic drugs are still needed, and the current EV purification methods limit the development of standardized and large-scale production [205].

Therapeutic effects of native EVs on different brain pathologies have been reported since 2011; most of them use EVs derived from mesenchymal stem cells (MSCs) for the treatment of stroke, traumatic brain injury, or AD. MSC-derived EVs show a homing mechanism toward injured brain tissue driven by inflammation. Other studies use EVs from neural stem cells (NSCs) isolated from mice or humans after the differentiation of induced pluripotent stem cells (iPSC), opening the perspective of a very personalized medicine by isolating iPSC from the patient himself. In this last case, the patient may benefit from his own cells after the generation of iPSCs [25,53]. NSCs-derived EVs demonstrate an outstanding innate tropism to make the brain capable of reaching the injury site [204]. In addition, dendritic cell-derived EVs have been proven to be promising for the treatment of brain cancers, which are resistant to immune cell recruitment, proposing them for immunotherapy against GBM [206].

To date, some EV-based treatments have undergone clinical trials:

- NCT03384433: EVs from allogenic placenta MSCs are IC injected to ameliorate the brain injury by promoting neurogenesis after an ischemic stroke [207];
- NCT05490173: MSC-derived EVs are intranasally administered to low-birth-weight infants to mitigate neurodevelopmental outcomes;
- NCT04202770: MSCs-derived EVs with transcranial focused US in patients with refractory, treatment-resistant depression, anxiety, and neurodegenerative dementia;
- NCT06138210: intravenous injection of EVs derived from human-induced pluripotent stem cells for ischemic stroke;
- NCT04388982: intranasal administration of allogenic adipose MSC-EVs in the treatment of mild to moderate dementia due to AD [208].

Besides the ongoing clinical trials, many other applications of EVs at the preclinical stage are listed in Table 4.

Although it has been demonstrated that EVs have clear potential for therapeutic applications already used in their native state, that is, being isolated from the cellular fluids in which they are dispersed after being secreted by a specific cell line, there are an increasing number of pharmaceutical applications of EVs engineered or functionalized after their isolation (Table 5).

### 5.5. Cell-Membrane-Derived Nanocarriers 

Cell-derived EV-mimetic nanocarriers have been used as an alternative to EVs, taking advantage of a much higher production yield for the drug delivery of different therapeutic molecules and NPs to the brain (Table 6).

The first membranes used were derived from red blood cells (RBCs), trying to exploit their prolonged circulating time due to the presence of membrane-oriented CD47. However, RBCs do not expose specific targeting, limiting their application to specific targets. 

Trying to achieve enhanced targeting ability, other sources of cell membranes have been evaluated to favor the therapeutic effects of the nanocarriers and their side effects. Hence, tumor cells, neutrophils, macrophages, and leukocytes, stem cells, natural killer cells, platelets, and bacteria have been assessed as cell membrane sources [312,313].

**Table 6:** Cell membrane-derived nanocarriers for the management of brain diseases.

Membranes’ Origin	Carrier	Cargo	Surface Functionalization	Disease	Administration Route	Reference
4T1 and platelet hybrid	Polymetformin + hyaluronic acid liposomes	Paeonol	-	Ischemic stroke	Intravenous	[314]
Aorta endothelial cells	HOP NPs	Rapamycin	CXCR4	Ischemic stroke	Intravenous	[315]
Brain microvasculature endothelial cells	Mesoporous silica NPs	Dihydroartemisinin	-	Cerebral malaria	Intravenous	[316]
	PLGA-PEG NPs	Doxorubicin	-	GBM	Intravenous	[317]
Dendritic cells	PLGA NPs	Rapamycin		Glioma	Intravenous	[318]
Macrophages	-	Molybdenum disulfide quantum dots	-	AD	Intravenous	[319]
	-	Cannabidiol	-	Post-traumatic stress disorder	Intravenous and US	[320]
	-	aPD-L1 and CXCL10	Angiopep-2	GBM	Intravenous	[321]
	Liposomes (DSPE-PEG2000)	IR-792	-	PTT of GBM	Intravenous	[322]
	Liposomes (DPPC, Chol, and DSPE-PEG2000)	Oxytocin	-	AD	Intranasal	[323]
	Mesoporous silica NPs	anti-NF-κB peptides	-	GBM	Intravenous	[324]
	Poly(N-vinylcaprolactam) nanogel	Manganese dioxide and cisplatin	-	Glioma	Intravenous	[325]
	Liposomes (Chol and soybean lecithin)	Baicalin	-	Ischemic stroke	Intravenous	[326]
	Cu_2−x_ Se and PVP NPs	Curcumin	DSPE-PEG2000-TPP	PD	Intravenous	[327]
	SLN (glycerol monostearate, Tween 80, and soya lecithin)	Genistein	RVG29 and TPP	AD	Intravenous	[328]
	PLGA	Rapamycin	PD-1	GBM	Intravenous	[329]
Microglia cells	Poly(propylene glycol dithiopropionate)	Zoledronate	-	GBM	Intravenous	[330]
	PLGA NPs	PLX3397	DSPE-PEG2000	Cognitive impairment	Intravenous	[331]
MSCs	Liposomes (PC)	Curcumin	-	Ischemic stroke	Intravenous	[332]
Monocytes	PLGA	Rapamycin	-	Ischemic stroke	Intravenous	[333]
Neutrophil	-	Fingolimod hydrochloride	-		Intravenous	[334]
	-	Mesoporous Prussian blue nanozyme	-		Intravenous	[335]
	PLGA NPs	Superparamagnetic iron oxide NPs	-	Neuroinflammation imaging	Intravenous	[336]
	Liposomes (DPPC + Chol + DSPE-PEG2000)	Leonurine	-	Ischemic stroke	Intravenous	[337]
	Dendrigraft poly-L-lysine and PEG NPs	Catalase	N-acetyl Pro-Gly-Pro		Intravenous	[338]
	β-cyclodextrin PBAP	Edaravone	SHp-PEG-DSPE		Intravenous	[339]
	PEI NPs	Octanoic acid	RVG29		Intravenous	[340]
Neural stem cells	-	Oncolytic adenovirus A4/k37	-	GBM	Intravenous	[341]
	Zein NPs	Antisense oligonucleotide	Aptamer 19S	PD	Intravenous	[342]
NK cells	PLGA NPs	Temozolomide and IL-15	cRGD peptide	GBM	Intravenous	[343]
Neuron cells	Cu2–xSe-PVP	Quercetin	VCAM-1	PD	Intravenous and US	[344]
Platelets	-	L-arginine and γ-Fe_2_O_3_ magnetic nanoparticles	-	Ischemic stroke	Intravenous	[345]
	T7-PEG-poly-histidine-poly-lysine	miRNA-Let-7c			Intravenous	[346]
	PLGA NPs	Human fat extract	RGD peptide		Intravenous	[347]
	Dextran NPs	Neuroprotectant (ZL006e)	Recombinant tissue plasminogen activator (rtPA) and thrombin-cleavable Tat-peptide		Intravenous	[348]
RBCs	-	Celecoxib	-	AD	Intranasal	[349]
	Mesoporous silica NPs + upconversion NPs	S-nitrosoglutathione	-	PD	Intravenous	[350]
	-	Doxorubicin	CDX peptide	Glioma	Intravenous	[351]
	-	Docetaxel nanocrystals	pHA-VAP peptide			[352]
	Surfactant	Docetaxel	cRGDyK peptide			[353]
	pH-sensitive NPs of acetal-dextran	Doxorubicin and lexiscan	Angiopep-2	GBM	Intravenous	[354]
	PEI + Poly-L-lysine NPs	siRNA			Intravenous	[355]
	Nanogel (Poly(deca-4,6-diynedioic acid) + Puilulan)	Temozolomide and indocyanine green	ApoE		Intravenous	[356]
	Acetal dextran	Temozolomide and OTX015			Intravenous	[357]
		ABT + A12 inhibitors			Intravenous	[358]
	NLC (Tween 80 + cetyl palmitate + oleic acid + chol + DSPE-PEG2000)	Resveratrol	RVG29 and TPP	AD	Intravenous	[359]
	Human serum albumin NPs	Curcumin	T807 and TPP		Intravenous	[360]
	-	Curcumin nanocrystals	RVG29	PD	Intravenous	[361]
	Boronic ester-Dextran	NR2B9C	Stroke-homing peptide	Ischemic stroke	Intravenous	[362]
	NLC (Chol oleate + Chol + soybean lecithin + triolein)	PARP inhibitor olaparib	C3 and SS31 peptides	Traumatic brain injury	Intravenous	[363]
Cancer cell-derived	PCL NPs	Indocyanine green	-	Fluorescent imaging and phototherapy of GBM	Intravenous	[364]
Brain cancer	Nanocomposite of PDPP3T + PLGA + PVA	Ultrasmall iron oxide NPs	cRGD peptide	Brain tumors	Intravenous	[365]
Breast cancer	PEG–PDPA	Succinobucol	-	Ischemic stroke	Intravenous	[366]
Brain metastatic breast cancer cell	mPEG-PLGA	Doxorubicin	-	Brain delivery	Intravenous	[367]
GBM cell line	pH-sensitive biomimetic NPs of acetal dextran	Temozolomide and cisplatin	-	GBM	Intravenous	[368]
	pH-sensitive polyglutamic acid	Doxorubicin	-		Intravenous + US	[369]
	PEI	pDNA (pHSVtk)	-		Intravenous/Intranasal	[370]
	Boron nitride nanotubes	Doxorubicin	-		Intravenous	[371]
	-	CuFeSe_2_ nanocrystals	-	Photothermal therapy GBM	Intravenous	[372]
	Nanosuspension	10-hydroxycamptothecin	-	Glioma	Intravenous	[373]
	PVP K30 + Sodium deoxycholate	Paclitaxel	WSW peptide		Intravenous	[374]
	Poly(MIs)-PEI	Paclitaxel	siPGK1	GBM	Intravenous	[375]
GBM from the patient	-	Au Nanorods	-	GBM	Intravenous	[376]
Glioma cell line	Cu_2−x_ Se NPs	Cinobufotalin	-	GBM	Intravenous	[377]
	Liposomes (DPPC, DSPC, DOPC, and Chol)	Indocyanine green	-	PTT of glioma	Intravenous	[378]
Metastatic melanoma	Citraconic anhydride grafted poly-lysine and polyethyleneimine xanthate	siPGK1	-	GBM	Intravenous	[356]
Brain metastatic breast cancer cells and glioma cells	Oleic acid, TPGS, and lanthanide-doped NPs	Gambogic acid and indocyanine green	-	Glioma	Intravenous	[379]
Dendritic cells and glioma cells	NE (lecithin)	Docetaxel	-	Glioma	Intravenous	[380]
GBM, macrophage, and microglia cells	Amphiphilic polymer chlorin e6, cucurbit[7]urils, and PEG	5-(3-methyltriazene-1-yl)imidazole-4-carboxamide	-	GBM	Intravenous	[381]
Mitochondria and GBM cells	PEG-PHB	Gboxin	-	GBM	Intravenous	[382]
Neutrophils and macrophages	PLGA NPs	Rapamycin	-	Glioma	Intravenous	[383]
Platelets and glioma cells	PLGA NPs	β-mangostin	-	Glioma	Intravenous	[384]
Platelets and RBCs	-	Hypoxia inducible factor-1α inhibitor YC-1	-	Ischemic stroke	Intravenous	[385]

CXCR4: C-X-C motif chemokine receptor 4; HOP: ROS-responsive amphiphilic copolymer HBA-OC-PEG2000; PBAP: phenylboronic acid pinacol ester; VCAM: vascular cell adhesion protein; pHA: p-hydroxybenzoic acid; PARP: poly(ADP-ribose) polymerase; PCL: polycaprolactone; PDPA: poly(2-(diisopropylamino)ethyl methacrylate; PVP: polyvinylpyrrolidone; PGK1: phosphoglycerate kinase-1; PEG-PHB: poly (ethylene glycol)-poly (4-(4,4,5,5-Tetramethyltetramethyl-1,3,2-dioxaborolan-2-yl) benzyl acrylate).

## 6. Conclusions

This review pointed out that nanomedicine is a significant tool for the development of efficient and safe brain disease treatments. The BBB, due to its structure, poses a formidable challenge to achieving effective drug delivery.

Nanosystems can be designed to reach the BBB and specifically deliver the cargo, thus increasing drug retention at the target site. For an efficacious design of drug delivery systems, a deep knowledge of the BBB structure and physiopathology is mandatory.

The lipid-based nanocarriers here described are versatile platforms, and several efforts have been made in the past years for the treatment of several BBB diseases.

Some liposomal formulations are currently used or are undergoing clinical trials, especially for tumor treatments. Some clinical trials are already active for the treatment of brain diseases with EVs. A relevant number of other nanocarriers were able to overcome the in vitro/vivo bottleneck since their activity was shown in animal models, paving the way for future clinical translation.

Nanotechnologies for therapeutic and diagnosis applications allow not only to enhance the availability of active anti-stroke, anticancer, antimicrobics, and neuroprotective agents for targeting e/o personalized brain drug delivery applications but also to empower the reengineering of a wide variety of small molecules and biologic drugs to assist neurodevelopment and face neurological diseases.

## Figures and Tables

**Table 1:** Liposomes for the treatment of brain diseases.

Composition	Drug	Surface Functionalization	Size (nm)	ZP (mV)	Disease	Administration Route	Reference
HSPC + DOPE + Chol + Didodecyldimethylammonium bromide	Doxorubicin	-	86 ± 2	−16.8 ± 1.4	Glioma	Intravenous + focused US	[115]
Egg lecithin + Chol + DSPE-PEG2000	Doxorubicin and carboplatin	-	212 ± 10	−13.0 ± 0.6	GBM	Intravenous	[116]
Lipoxal^TM^	Oxaliplatin	-	-	-	GBM	CED	[117]
DPPC + Chol + poloxamer L64	Oligonucleotide	-	100–200	-	Spinocerebellar ataxias	Intravenous	[118]
Ionizable lipid + DSPC + Chol + PEG2000-DMG	siRNA	-	-	-	Polyglutamine diseases (HD)	ICV	[119]
DPPC + Chol + PEG2000	Pramipexole	-	122 ± 0.13	−10.60 ± 0.12	PD	Intraperitoneal	[120]
Soybean PC + Chol + DSPE-PEG2000	Nicotinamide riboside	-	152	−22	Ischemic stroke	Intravenous	[121]
POPS + phosphatidylserine	Mesoporous silica NPs and phospholipase A2	-	<200	<−30	Detoxification	Intravenous	[122]
POPC + Chol + DSPE-PEG2000 + dimyristoyl-phosphatidic acid	Pep63	Tf	132 ± 22	−16.5 ± 0.9	AD	Intravenous	[123]
Soy PC + Chol + DSPE-PEG2000	Dopamine HCl		182 ± 8	+7.5 ± 1.2	PD	In vitro model (transwell)	[124]
DSPC + Chol + POPG + DSPE-PEG2000	Temozolomide and bromodomain inhibitor JQ1		137	−12	GBM	Intravenous	[125]
EPC + Chol + DSPE-PEG2000-MAL	Osthole		104 ± 4	−7.0 ± 0.6	AD	Intravenous	[126]
Soy lecithin + Chol + DSPE-PEG2000	Elemene and cabazitaxel	Tf and cell membrane proteins	135 ± 4	+33.6 ± 0.7	Glioma	Intravenous	[127]
DOTAP + DOPE	Temozolomide	Anti-TfR single-chain Ab fragment	41 ± 9	+30 ± 5	GBM	Intravenous	[128]
	SOD1 siRNA		~100	-	Neuroinflammation and apoptosis	Intravenous	[129]
Chol + sphingomyelin + DSPE-PEG2000-MAL	Doxorubicin	Anti-Tf mAb (MYBE/4C1)	142 ± 4	−18 ± 4	Crossing the BBB	In vitro model (transwell)	[130]
POPC + DOTAP + DSPE-PEG2000	Dopamine	Anti-Tf OX-26 mAb	~85	-	PD	Internal carotid artery perfusion	[131]
DSPC + Chol + DSPE-PEG2000	Oxaliplatin		139.3 ± 1.5	−21.9 ± 1.0	Brain delivery	Intravenous	[132]
PC + Chol + DSPE-PEG2000-MAL (+ DPPG)	Cis-diamminedinitratotplatinum(II)	Anti-VEGFR and Anti-VEGFR 2 mAb	126 ± 10 (136 ± 11) in PBS 143 ± 12 (−26 ± 4) in H_2_O	−1.6 ± 0.3 (−7.6 ± 1.1) in PBS 158 ± 13 (−39 ± 5) in H_2_O	GBM	Intravenous	[133]
DC + Chol + DOPE + DSPE-PEG2000	Paclitaxel and survivin siRNA	Anti-CD133 aptamer and angiopep-2	119 ± 6 in H_2_O	11.5 ± 0.6 in H_2_O	GBM	Intravenous	[134]
DPPC + Chol + DSPE-PEG2000	Magnetic NPs and camptosar	Cetuximab	194 ± 2	+2.3 ± 0.1	Glioma	Intravenous and alternating magnetic fields	[135]
DOPC + Chol + DSPE-PEG2000	miRNA-92b inhibitor	ApoE	41 ± 6	−3 ± 3	GBM	Intravenous	[136]
DMPC + Chol	Porphyrin	ApoE3	29 ± 9	-	GBM	Intravenous	[137]
EPC + DOTAP + Chol + DSPE-PEG2000	Doxorubicin	CPP R8	95 ± 5	+12 ± 4	Glioma	Intravenous	[138]
Soy PC + Chol + DSPE-PEG2000	Paclitaxel	R8-dGR peptide	100–120	-	Glioma	Intravenous	[139]
DSPC + DPPC + Chol + Cardiolipin + phosphatidic acid + DSPE-PEG2000	Nerve growth factor, rosmarinic acid, curcumin, and quercetin	CPP TAT peptide	159	−28	AD	Intravenous	[140]
DOTAP + DOPE + DSPE–PEG2000	ApoE2 encoding plasmid DNA	CPP RVG and mannose	168 ± 3	+20 ± 4	AD	Intravenous	[141]
		CPP, penetratin, and mannose	172 ± 3	+19.0 ± 0.9			
Lipoid S100 + Chol + mPEG2000-DSPE	N-3,4-bis(pivaloyloxy)-dopamine	RVG29	135 ± 3	−14 ± 0.4	PD	Intravenous	[142]
DSPC + DPPC + Chol + Cardiolipin + dihexadecyl phosphate + DSPE-PEG2000	Ceftriaxone, FK506, and nilotinib	GSH	160	−39	PD	In vitro model (transwell)	[143]
DSPC + Chol + DSPE-PEG2000	Gefitinib	GSH and Tween 80	86 ± 4	−3.8 ± 0.9	Glioma	In vitro model (transwell)	[144]
		α-helical CPP	147 ± 4	−1.7 ± 0.2			
Soybean PC + Chol + DSPE-PEG2000	Paclitaxel	CPP dNP2 and folic acid	104	−6	Glioma	Intravenous	[145]
DSPC + Chol + POPG + DSPE-PEG2000	Temozolomide and bromodomain inhibitor JQ1	Folate	165	−14	GBM	Intravenous	[125]
Soy PC + Chol	Paclitaxel	Vitamin C and glucose	109 ± 3	−4.5 ± 0.5	Glioma	Intravenous	[146]
	Resveratrol	Vitamin E, TPGS	65 ± 6	−1.1 ± 1.1	Glioma	Intravenous	[147]
DPPC + Chol	Docetaxel and quantum dots	RGD, and vitamin E TPGS	182 ± 8	+1.1 ± 0.3	Glioma	Intravenous	[148]
	Docetaxel AuNPs with glutathione	Vitamin E and TPGS and TfR	268 ± 10	−6 ± 5	Crossing the BBB	Intravenous	[149]
DSPC + Chol + DMPC + phosphatidylserine	astragaloside IV and nestifin-1	Wheat germ, agglutinin, and leptin	Many	-	PD	In vitro model (transwell)	[150]
EPC + Chol + DSPE-PEG2000	Curcumin and quinacrine	Mannose	119.7 ± 0.2	−2.7 ± 0.7	GBM	IC	[151]
C12-alkyl-mannopyranoside + Chol	Dynantin		232 ± 4	-	Depression	Intranasal	[152]
EPC + Ginsenoside Rh2 + Chol + DSPE-PEG	Paclitaxel	Menthol	102 ± 7	11.7 ± 0.1	GBM	Intravenous	[153]
EPC + Chol + DSPE-PEG2000	Daunorubicin	PEI and 4-Aminophenyl β-D-glucopyranoside	106 ± 3	−8.7 ± 0.4	Glioma	Intravenous	[154]
EPC encapsulating PLGA NPs	Rivastigmine	Dextran and cholic acid	112 ± 11	-	AD	Intravenous	[155]

ZP: zeta-potential; DOPE: dioleoylphosphatidylethanolamine; DPPC: dipalmitoylphosphatidylcholine; PEG2000-DMG: 1,2-dimyristoyl-rac-glycero-3-methoxypolyethylene glycol-2000; POPS: 1-palmitoyl-2-oleoyl-sn-glycero-3-phosphoserine; POPC: 1-palmitoyl-2-oleoyl-glycero-3-phosphocholine; POPG: 1-palmitoyl-2-oleoyl-sn-glycero-3-phospho-(1′-rac-glycerol); MAL: maleimide; DOTAP: 1,2-dioleoyl-3-trimethylammonium-propane; PC: phosphatidylcholine; PLGA: poly(lactic-co-glycolic acid); TPGS: D-α-tocopheryl polyethylene glycol succinate; PEI: polyethylenimine.

**Table 2:** SLN for the treatment of brain diseases.

Composition	Drug	Surface Functionalization	Size (nm)	ZP (mV)	Disease	Administration Route	Reference
Compritol 888 ATO + stearic acid + span 60	Levofloxacin and doxycycline	-	∼50	-	Bacterial infection	Intranasal	[159]
Witepsol E 85	RVG-9R and BACE1 siRNA	Chitosan	358 ± 26	+10.5 ± 0.8	AD	Intranasal	[160]
Cetyl palmitate + Tween80	Quercetin	Tf	234 ± 18	−32 ± 8	AD	In vitro model (transwell)	[161]
Sodium behenate + sodium stearate + PVA120000 + PEG	Methotrexate		500 ± 45	-	GBM	Intravenous	[162]
Glyceryl monostearate + stearic acid + soy lecithin	Docetaxel	Lf	121 ± 6	−21.5 ± 1.2	Glioma	Intravenous	[163]
Behenic acid + tripalmitin + cacao butter + DSPE-PEG(2000)	Tamoxifen and carmustine		Many	Many	GBM	In vitro model (transwell)	[164]
Sodium behenate + sodium stearate + PVA120000 + PEG	Methotrexate	Insulin	445 ± 41	-	GBM	Intravenous	[162]
Dynasan 116 + Tween80	Donepezil	ApoE	147.5 ± 0.8	−9.6 ± 0.5	AD	In vitro model (transwell)	[165]
Lecithin soya + stearic acid+ Tween80	Docetaxel	Angiopep-2	111 ± 3	−16.4 ± 1.2	GBM	Intravenous	[166]
Cetyl palmitate + Tween80	Resveratrol and grape skin/seeds	Anti-Tf OX-26 mAb	254 ± 17	−4.0 ± 0.1	AD	In vitro model (transwell)	[167]
Chol + sphingomyelin + phosphatidylserine + sphingosine + phosphatidylethanolamine	Methylprednisolone	Anti-contactin-2 mAb	158 ± 19	−8.7 ± 0.5	Multiple sclerosis	Intravenous	[168]
		Anti-neurofascin mAb	162 ± 13	−8.7 ± 0.4			

PVA: polyvinyl alcohol.

**Table 4:** Native EVs for the treatment of brain diseases.

Origin	Mechanism of Action	Disease	Administration Route	References
Astrocytes	Ameliorated neuronal damage through regulating autophagy	Ischemic stroke	Intravenous	[209]
Embryonic stem cells	Promote neurological recovery	Ischemic stroke	Intravenous	[210]
MSCs	β-amyloid degradation, immunoregulation, and neurotrophic action	AD	IC	[211]
	Protect neurons against amyloid-β peptide-induced oxidative stress and synapse damage		In vitro model (transwell)	[212,213]
	Immunomodulatory and neuroprotective effects		Intranasal	[214,215,216]
	Neuroprotective and reduce neuroglia activation	Amyotrophic lateral sclerosis	Intranasal	[217]
	Behavioral improvement	Autism	Intranasal	[218,219,220]
	Diminished loss of glutamatergic and GABAergic neurons, reduced inflammation, neuroprotective, and anti-inflammatory effects	Epilepsy	Intranasal	[221]
	Reduced neuronal apoptosis and improved neurological function	Hemorrhage	Intravenous	[222]
	Reduce the infarct zone, favor neurological and functional recovery, and promote neurovascular remodeling	Ischemic stroke	Intravenous	[223,224]
	Promote neurogenesis and angiogenesis		Intranasal	[225,226]
	Enhanced angiogenesis	PD	Intraperitoneal	[227]
	Reduced microglia-mediated neuroinflammation	Perinatal brain injury	Intranasal	[228]
	Reduce glutamate levels and preserve the number of parvalbumin-positive GABAergic interneurons	Schizophrenia	Intranasal	[229]
	Increase newborn ECs, reduce neuroinflammation, promote angiogenesis and neurogenesis, decrease neuron cell death, and inhibit ferroptosis	Traumatic brain injury	Intravenous	[230,231,232,233]
Microglia cells	Attenuate brain injury and promote neural survival	Ischemic stroke	Intravenous	[234]
Neural stem cells	Neuroprotective, reduce edema, protect astrocytes, and reduce infarct volume	Ischemic stroke	Intravenous	[235,236,237]
	Neurological recovery and neuroregeneration in mice		Internal carotid artery perfusion	[238]
	Reduced lesion volume and microgliosis, improved spontaneous movements, and increased neuronal survival		ICV	[239]
	Neuroprotection	AD	Intravenous	[240]
T cells	Reduces pro-inflammatory transcripts and neuroinflammatory responses, slowing disease progression	Amyotrophic lateral sclerosis	Intranasal	[241]
Ginseng	Inhibit glioma progression and regulate tumor-associated macrophages	Glioma	Intravenous	[242]
*Escherichia coli*	Antitumor effect	Neuroblastoma	Intravenous	[243]
*Lactobacillus plantarum*	Reduced apoptosis in ischemic neurons	Ischemic stroke	ICV	[244]
	Increased BDNF expression in the hippocampus produces antidepressant effects	Depression	Intraperitoneally	[245]

**Table 5:** Engineered EVs for the management of brain diseases.

Origin	Drug	Surface Functionalization	Disease	Administration Route	Reference
Astrocytes	Homer1	-	Hemorrhage	IC	[246]
	Ultrasmall superparamagnetic NPs	-	Brain delivery	Intranasal	[247]
	miR-143-3p	-	Intracerebral hemorrhage	Intravenous	[248]
Blood	Dopamine	Tf	PD	Intravenous	[249]
Brain endothelial cells	VEGF siRNA	-	Brain cancer	Intravenous	[250]
	TPP-Ce6	Saturated TfR	GBM	Intravenous and light	[251]
Dendritic cells	VEGF-A siRNA and doxorubicin	-	Glioma	Intranasal	[252]
	Curcumin and siSNCA in polymeric NPs	RVG peptide	PD	Intravenous	[253]
	Short hairpin RNA microcircles			Intravenous	[254]
Embryonic stem cells	Curcumin	-	Ischemic stroke	Intranasal	[255]
	Paclitaxel	c(RGDyK) peptide	GBM	Intravenous	[256]
Endothelial progenitor cells	miR-126	-	Ischemic stroke	Intravenous	[257]
Fibroblasts	Achaete-scute homolog 1, myelin transcription factor 1 like, and POU-III transcription factor Brain-2	Metabotropic glutamate receptor 8	Brain delivery	Intranasal	[258]
	Methotrexate	KLA-LDL peptide	GBM	Intravenous	[259]
HEK-293T cells	miR-21-sponge	-	GBM	Intratumor	[260]
	Doxorubicin	Angiopep-2 and TAT peptides	Glioma	Intravenous	[261]
	Verrucarin A	EGFR mAb	GBM	Intravenous	[262]
	AMO-21	Lamp2b-T7	GBM	Intravenous	[263]
	CircDYM	Lamp2b-RVG	Depressive disorders	Intravenous	[264]
	Aptamer F5R2	RVG peptide	PD	Intravenous	[265]
	circSCMH1		Ischemic stroke	Intravenous	[266]
	Nerve growth factor			Intravenous	[267]
	mRNA SNAP25 and Gap43		AD	Intravenous	[268]
	Single guide RNA and dCas9-DNMT3A		PD	Intravenous and US	[269]
Hippocampal cells	Adenosine	-	Ischemic stroke	Intravenous	[270]
Leukocytes	Retrovirus-like mRNA-packaging capsids	-	Brain delivery	Intravenous	[271]
Macrophages	TPP1	-	Batten disease	Intraperitoneal	[272]
	Curcumin		AD	Intravenous	[273]
	BDNF	-	Inflammation	Intravenous	[274]
	GDNF	-	PD	Intranasal	[275]
	Recombination signal-binding protein-Jκ	-	Glioma	Hypodermically injected	[276]
	SPIONs and curcumin	Neuropilin-1-targeted peptide		Intravenous	[277]
Macrophages and blood serum	Doxorubicin	-	Glioma	Intravenous and US	[278]
MSCs	-	RVG	AD	Intravenous	[279]
	-	AAV capsid-specific peptides- Lamp2b	Brin delivery	Intravenous	[280]
	Neprilysin	-	AD	Intranasal	[281]
	BDNF	-	Ischemic stroke	Intranasal	[282]
	miR-126	-		Intravenous	[283]
	Magnetic iron oxide NPs	-		Intravenous	[284]
	Antisense oligonucleotide 4	-	PD	ICV	[285]
	miR-124	-	Traumatic brain injury	Intravenous	[286]
	miR-29a-3p	-	Glioma	Intravenous	[287]
	miR-133	-	IC hemorrhage	Intravenous	[288]
	Curcumin	c(RGDyK) peptide	Ischemic stroke	Intravenous	[289]
	miR-210	Lamp2b-RVG		Intravenous	[290]
	miR-124			Intravenous	[291]
	Curcumin and SPIONs	Penetratin and RVG29	PD	Intranasal	[292]
Microglia cells	-	DA7R and SDF-1	Ischemic stroke	Intravenous	[293]
	lincRNA-Cox2	-	Lipopolysaccharide-induced microglia proliferation	Intranasal	[294]
	miR-124-3p	-	Traumatic brain injury	Intravenous	[295]
	NR2B9c	RVG29		Intravenous	[296]
			Ischemic stroke	Intravenous	[297]
	Doxorubicin	Amphiphilic peptide	GBM	Intravenous	[298]
Neural progenitor cells	-	RGD-4C peptide	Ischemic stroke	Intravenous	[299]
	PD-L1 siRNA	c(RGDyK) peptide	GBM	Intravenous and radiation	[300]
Neural stem cells	Bryostatin-1	Ligand of PDGFRα	MS	Intravenous	[301]
	Anti-miRNA-21 and miRNA-100	CXCR4	GBM	Intranasal	[302]
Neutrophils	Doxorubicin	-	Glioma	Intravenous	[303]
Plasma	Donepezil		AD	Intravenous	[304]
	Tf	-	MS	Intranasal	[305]
	Quercetin	-	AD	Intravenous	[306]
		mAB Gap43	Ischemic stroke	Intravenous	[307]
Grapefruit	Doxorubicin	Heparin and cRGD	Glioma	Intravenous	[308]
	miR-17	Folic acid	GBM	Intranasal	[309]
*Escherichia coli*	Pioglitazone	-	Ischemic stroke	Intravenous	[310]
*Salmonella*	Doxorubicin	-	Glioma	Intravenous	[311]

TPP: triphenylphosphonium; Ce6: chlorin e6; siSNCA: siRNA targeting SNCA; SPIONs: superparamagnetic iron oxide nanoparticles; AAV: adeno-associated virus; Lamp2: lysosomal-associated membrane protein 2; BDNF: brain-derived neurotrophic factor; GDNF: glial-cell-line-derived neurotrophic factor.
